# The First Moroccan Experience of Personalized Template‑Guided High-Dose-Rate Brachytherapy for Organ Preservation in Glans Squamous Cell Carcinoma: Game Changer or Challenge?

**DOI:** 10.7759/cureus.108660

**Published:** 2026-05-11

**Authors:** Hamza Samlali, Najlaa Assaid, Youness Khobbaizi, Redouane Samlali, Hassan Jouhadi

**Affiliations:** 1 Radiation Oncology Department, Littoral Clinic - Oncorad Group, Casablanca, MAR; 2 Research Department, Littoral Clinic - Oncorad Group, Casablanca, MAR; 3 Medical Physics Department, Littoral Clinic - Oncorad Group, Casablanca, MAR; 4 Radiation Oncology, University Hospital Center Ibn Rochd, Faculty of Medicine and Pharmacy, Hassan II University, Casablanca, MAR

**Keywords:** glans squamous cell carcinoma, high-dose-rate brachytherapy, organ preservation, penile cancer, personalized template

## Abstract

Squamous cell carcinoma of the glans is a rare malignancy, often diagnosed at an advanced stage, and its management remains challenging. Therapeutic approaches are multimodal and depend on tumor stage, risk of recurrence, nodal involvement, and disease extent. Organ-preserving strategies are increasingly advocated, particularly in patients who refuse mutilating surgery.

We report the first Moroccan case of a 50-year-old man diagnosed with a T2N0M0 glans carcinoma, treated with high-dose-rate (HDR) brachytherapy using a customized template following refusal of partial penectomy. Initial treatment achieved adequate tumor coverage without acute toxicity, with no evidence of necrosis or skin ulceration. A superficial recurrence in the previously irradiated area was detected four months later and was successfully treated with salvage HDR brachytherapy. At 18 months of follow-up, urinary function was preserved and partial sexual function maintained, with no severe complications.

This case illustrates that personalized HDR brachytherapy may represent a feasible, safe, and organ-preserving alternative in patients refusing mutilating surgery, supporting its applicability even in resource-limited settings.

## Introduction

Squamous cell carcinoma of the penis (SCCP) is a rare malignancy with a highly variable incidence worldwide [1]. In high-income countries, its incidence is estimated at approximately one per 100,000 men per year, with the vast majority being squamous cell carcinomas [2]. In contrast, in several regions of Africa, Asia, and South America, the incidence of penile cancer is significantly higher than in high-income countries. Owing to its rarity, available data remain insufficient to clearly establish its pathogenesis. SCCP may arise from malignant transformation of penile intraepithelial neoplasia or develop de novo, often in association with known risk factors such as human papillomavirus (HPV) infection, phimosis, lack of circumcision, chronic inflammatory conditions including balanoposthitis, and tobacco use [3,4].

In low-resource settings, delayed diagnosis is common, with a predominance of locally advanced stages that limit organ-preserving therapeutic options.

Localized SCCP can be effectively managed with various local treatments including topical therapy, surgery, or radiotherapy. Because of early lymphatic spread and the limited ability of imaging to detect micrometastases, early surgical staging of inguinal lymph nodes remains essential. Advanced disease often requires multimodal management, although the optimal therapeutic sequence is yet to be defined. Treatment selection depends on tumor size, stage, grade, histology, anatomical location, and patient preference [5]. The standard treatment for localized penile cancer is usually partial or total penectomy, which provides good local control with high five-year survival rates in early-stage disease. However, these procedures are often associated with significant urinary, sexual, and psychological morbidity, which may limit their acceptability for some patients.

Brachytherapy represents an effective organ-preserving radiotherapeutic modality. It consists of placing radioactive sources directly within or in close proximity to the tumor, allowing delivery of a high dose to the target while minimizing exposure to surrounding healthy tissues. This approach differs from external beam radiation therapy (EBRT), which delivers radiation through intervening tissues, and from low-dose-rate (LDR) or pulse-dose-rate (PDR) brachytherapy, which deliver radiation continuously or in pulses over a prolonged period. High-dose-rate (HDR) brachytherapy, in particular, offers superior dose distribution and improved radioprotection [6]. Several studies from European and North American centers have reported the use of both LDR and HDR brachytherapy for localized penile cancer, demonstrating favorable local control and high rates of organ preservation [2,7,8]. Despite these advantages, HDR brachytherapy remains underutilized in routine clinical practice, mainly due to its technical complexity, high cost, and the limited availability of specialized centers. This contributes to the scarcity of published data from low- and middle-income countries. However, these experiences remain largely confined to high-income settings, and access to HDR brachytherapy is still limited in many low- and middle-income countries due to technical, economic, and expertise-related constraints.

To our knowledge, no published case of HDR brachytherapy for penile cancer has been reported to date in Africa or Morocco.

We report here the first known Moroccan case of penile cancer treated with HDR brachytherapy, performed in a private radiotherapy center. This work aims to describe the technical workflow, early clinical outcomes, and the potential role of this approach in low-resource environments.

## Case presentation

We report the case of a 50-year-old man with no significant medical history, a former smoker and alcohol consumer who had quit in 2006. He initially presented with dysuria associated with urethral stenosis. Clinical examination revealed a 2-cm lesion on the ventral aspect of the glans, encircling the urethral meatus and extending dorsally along a downward trajectory toward the frenulum, while remaining anterior to the coronal sulcus. A superficial satellite nodule measuring 5 mm was also identified at the ventral-dorsal junction of the glans at the 9-o’clock position relative to the peri-meatal lesion. PET-CT revealed no locoregional or distant disease. 

Initial evaluation showed high-grade squamous intraepithelial neoplasia of the urethra, associated with HPV-related condylomatous lesions, confirmed by biopsy. A second biopsy revealed a differentiated, non-keratinizing, infiltrating squamous cell carcinoma, staged clinically as T2N0M0 [9].

Several well-established risk factors were present, including HPV infection associated with condylomatous lesions and a history of prolonged tobacco use. The progression from intraepithelial neoplasia to invasive carcinoma is consistent with malignant transformation in a high-risk setting. Multidisciplinary discussion recommended glansectomy as standard of care, but the patient refused categorically. An organ-sparing approach using HDR brachytherapy was therefore proposed, and informed consent was obtained after detailed counseling on risks and benefits.

**Figure 1:**
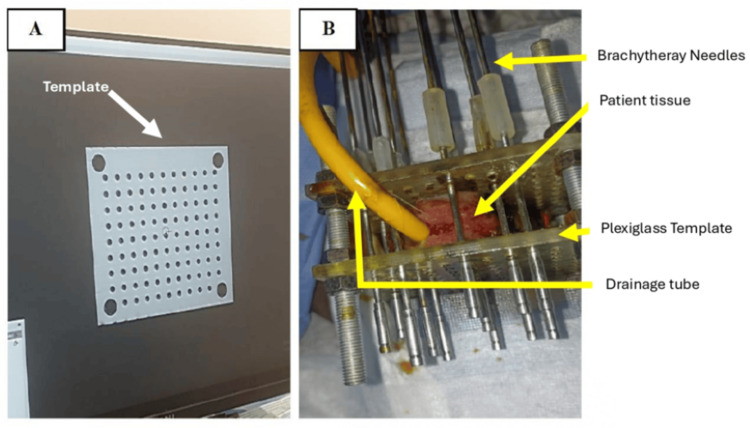
(A) 3D model of the needle placement template used for planning high-dose-rate (HDR) brachytherapy of the penis; (B) Intraoperative view of the brachytherapy template with needles positioned in the penis during the procedure. Needles placement and treatment data are from the present study; the design of the template was based on Parsai et al., 2018 [10].

For interstitial brachytherapy planning, a personalized template was created using selective deposition lamination rapid prototyping, allowing precise 3D reconstruction of the patient’s anatomy (Figure 1A). The three-dimensional printed template was then used to guide accurate needle implantation into the glans during the procedure (Figure 1B). The device consisted of a 6.5 × 5-cm rectangular grid made of plexiglass, with 1-mm cylindrical holes spaced 5 mm apart, ensuring parallel and uniformly spaced needle placement.

The catheters were placed in a parallel fashion approximately 1 cm apart, following the methodology described by Parsai et al. Special care was taken to avoid urethral penetration, aided by urethral catheterization for identification during implantation [10]. In addition, a superficial satellite nodule was treated using a single pleiobrachytherapy catheter, as seen in Figure 1B. A preliminary dosimetric simulation optimized dose distribution to the target while sparing organs at risk. This device was developed in-house and did not rely on any proprietary software.

**Figure 2:**
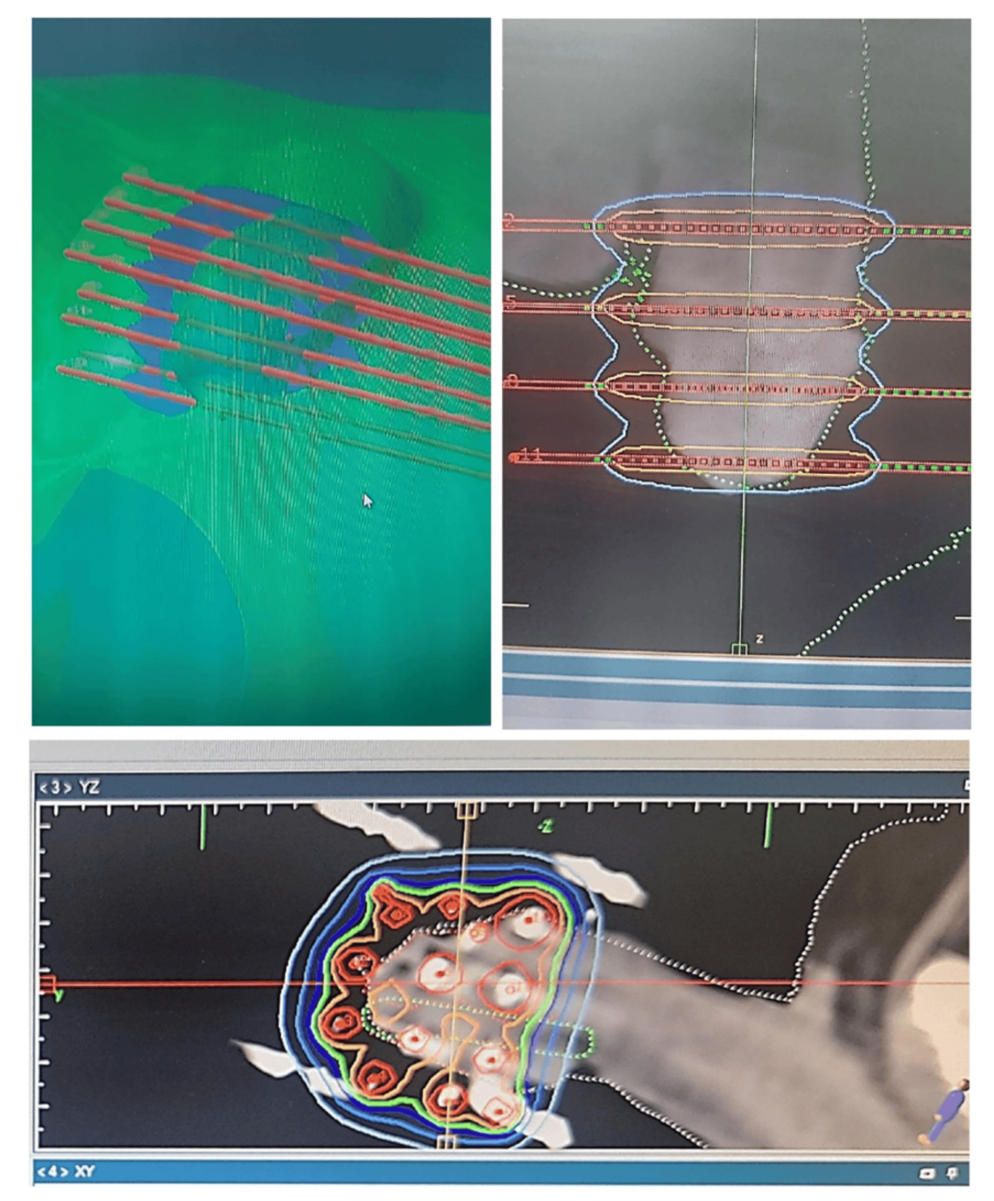
Treatment planning in penile brachytherapy. (A) 3D representation of needle implantation showing the trajectories of the radioactive sources. (B) Longitudinal view with needle reconstruction and isodose distribution adapted to the implant geometry. (C) Axial view of the treatment plan demonstrating isodose coverage of the target volume while sparing surrounding healthy tissues.

The first HDR brachytherapy course was performed under spinal anesthesia, with implantation of 14 needles using the plexiglass template (Figure 2). CT-based planning with SagiPlan software (Elekta AB, Stockholm, Sweden; proprietary licensed software) delivered 10 fractions of 2.5 Gy twice daily over five days (total dose 25 Gy). Local care was provided between applications to prevent maceration around the glans and coronal sulcus. Dosimetric parameters - D90 2.5 Gy (100.7% of prescription), V100 92.7%, and conformity index (COIN) 0.403 - reflected satisfactory target coverage and acceptable dose to organs at risk (Table 1) [11]. Dose constraints for organs at risk were applied according to GEC-ESTRO/ABS recommendations [12]. For the urethra, D2cc and V115 were carefully monitored to minimize exposure while ensuring adequate target coverage. For the superficial tissues of the glans and prepuce, the maximum point dose per fraction was limited to 120% of the prescribed fraction dose, resulting in a cumulative dose across all fractions kept below 32 Gy, in line with published recommendations by Crook et al., 2013 [12].

**Table 1:** Dosimetric Parameters of Initial and Salvage High-Dose-Rate (HDR) Brachytherapy Treatments This table presents the main dosimetric parameters for both the initial and salvage HDR brachytherapy treatments, demonstrating adequate target coverage and acceptable doses to organs at risk. Data are derived from the present study. Dosimetric indices and treatment planning were performed according to Baltas D and Zamboglou N (2006) [11]. Abbreviations: V100 = volume receiving 100% of the prescribed dose; V150 = volume receiving 150% of the prescribed dose; D90 = dose delivered to 90% of the target volume; D2cc = dose to the most exposed 2 cc of the organ at risk; COIN = Conformity Index. D90 (Gy): expressed as both absolute dose and percentage of the prescribed dose. D2cc urethra (Gy): maximum dose delivered to the most exposed 2 cc of the urethra.

Parameter	1st Brachytherapy	2nd Brachytherapy (Salvage)
Number of implanted needles	14	15
Number of fractions	10	8
Dose per fraction (Gy)	2.5	2.5
Total dose (Gy)	25	20
V100 (%)	92.2	95.2
V100 (cc)	16.7	20.7
D90 (Gy)	2.5 (100.7%)	2.6 (103.9%)
V150 (%)	33.6	21.3
V150 (cc)	6.2	4.6
Conformity Index (COIN)	0.351	0.335
D2cc urethra (Gy)	3 (121%)	2.3 (31.6%)
Urethra V115 (%)	2.4 cc (69.4%)	0 cc (0.8%)
Irradiation time	06 min 25 s	08 min 22 s
Total treatment duration	5 days	5 days
Immediate complications	None	None

No acute toxicity was observed, with no evidence of tissue necrosis, ulceration, or significant local complications.

Follow-up showed good tolerance, with no dysuria or hematuria. A limited local recurrence on the prepuce (1.5 × 1 cm) was detected within the previously irradiated area four months after the initial HDR brachytherapy course. A second salvage HDR brachytherapy procedure was performed with 15 implanted needles, delivering eight fractions of 2.5 Gy (total 20 Gy). Dosimetric parameters remained acceptable (V100 95.2%, D90 103.9%), despite a slight decrease in COIN (0.335) (Table 1). Clinical evolution remained favorable with preserved urinary function. The patient was followed for 18 months after the initial HDR brachytherapy, during which urinary function remained normal, with no evidence of meatal or urethral stenosis.

This case report did not require institutional review board approval according to our institutional policies. Written informed consent for treatment and publication was obtained from the patient.

## Discussion

Glans squamous cell carcinoma is a rare malignancy, accounting for less than 1% of all male cancers in high-income countries, with an annual incidence of approximately one per 100,000 men [13]. Its true incidence in low-resource settings is likely underestimated due to the lack of screening programs, sociocultural barriers, and limited access to specialized care [14]. To our knowledge, this is the first reported case of HDR brachytherapy for penile localization in Morocco and in Africa.

This case illustrates several clinical and therapeutic challenges in managing an infiltrating squamous cell carcinoma in a relatively young patient with HPV-related condylomatous lesions. The glans is the most frequently affected site in SCCP, followed by the prepuce, coronal sulcus, and penile shaft [6]. Well-documented risk factors were present, including HPV infection and a history of prolonged tobacco use, both established contributors to SCCP pathogenesis [3].

Although current guidelines recommend partial penectomy as the standard initial treatment for T2 lesions to ensure optimal local control [5,15], the patient’s categorical refusal necessitated a conservative approach. Historically, surgery - partial or total penectomy - has been the gold standard, particularly for advanced stages, due to its effectiveness in local control. However, these interventions are associated with significant morbidity, including adverse effects on sexual function, body image, and overall quality of life [16]. Consequently, organ-preserving strategies have been developed and are now preferred in early-stage disease or in patients refusing mutilating surgery. Among these, external beam radiotherapy (EBRT) and brachytherapy are validated alternatives [6], with EAU-ASCO guidelines recognizing radiotherapy as a conservative treatment option for T1a, T1b, and selected T2 penile tumors [5].

Retrospective studies suggest that EBRT yields inferior outcomes compared with brachytherapy in terms of five-year recurrence-free survival and organ preservation [17]. HDR brachytherapy, chosen in this case due to the patient’s refusal of mutilating surgery, represents a feasible, organ-sparing strategy in experienced centers, and is mentioned in international guidelines for carefully selected T1-T2 lesions measuring less than 4 cm. Precision and reproducibility in this anatomically complex region were achieved via a personalized rapid-prototyped template, optimizing needle placement and dose homogeneity. Treatment planning required meticulous dosimetric calculations to ensure target coverage and organ-at-risk sparing, highlighting the technical and multidisciplinary effort necessary for this individualized approach.

The patient, staged T2N0M0 with a 2-cm lesion, no corporal involvement, and no regional or distant spread, initially received a glansectomy recommendation per guidelines. After patient refusal, a multidisciplinary consensus led to HDR brachytherapy as a conservative alternative. Dosimetric planning achieved satisfactory target coverage (V100 > 90%, D90 > 100%). A superficial preputial recurrence occurred four months later, consistent with the reported recurrence rates of ~15-25% in HDR cohorts and up to ~30% in pooled series. Contributing factors may include tumor aggressiveness, intrinsic radioresistance, and inability to perform standard surgery [18]. According to current guidelines, salvage surgery would be appropriate; however, in light of a second refusal, a second course of HDR brachytherapy was successfully delivered.

According to the EAU systematic review, five-year local control rates (recurrence-free survival, RFR) after brachytherapy range from 50% to 67% for T2 tumors, with an overall mean of 78.6% across all stages. By comparison, surgery provides slightly higher local control rates (76-84%), although no significant difference in overall survival has been demonstrated. Penile preservation is achieved in approximately 74% of cases treated with brachytherapy, with acceptable morbidity and generally satisfactory urinary and sexual outcomes [5].

In our case, the first HDR brachytherapy course achieved satisfactory target coverage (V100 > 90%, D90 > 100%). Four months later, a superficial recurrence was observed in the prepuce, consistent with reported HDR recurrence rates (~15-25% in cohorts, up to ~30% in pooled series [19]). This recurrence may reflect tumor aggressiveness, intrinsic radioresistance, inability to perform standard surgery, or a second primary lesion within the context of HPV-associated field cancerization. Notably, the recurrence occurred in an area prescribed a slightly lower dose to reduce urethral or skin toxicity, highlighting the delicate balance between tumor control and organ preservation, and underscoring the complexity of individualized HDR brachytherapy planning.

HDR brachytherapy can be associated with specific complications, including urethral stenosis (20-35%), glans necrosis (10-20%), corporal fibrosis, and meatal stenosis (historically up to 40%, now reduced to 6.6% in contemporary series) [5]. No severe complications were observed in our patient, who maintains normal urinary function and partially preserved sexual activity, aligning with literature reporting ~70% satisfactory sexual function post-brachytherapy [5]. 

Brachytherapy, particularly HDR brachytherapy, is increasingly recognized as a conservative organ-preserving treatment option for early-stage penile cancer. Existing literature supports its role as a conservative approach without compromising oncological outcomes. A meta-analysis by Hasan et al. (2015) demonstrated comparable five-year overall survival between patients treated with penectomy (76%) and those treated with brachytherapy (73%), with similar local control for T1/T2 lesions. The organ preservation rate with brachytherapy was approximately 74%, and most recurrences were successfully managed surgically, indicating that initial conservative management does not preclude definitive intervention if needed. These findings are consistent with our case, in which HDR brachytherapy allowed preservation of the penis as well as maintenance of urinary and partial sexual function in a patient who refused mutilating surgery [20].

Additional evidence comes from a recent multicenter French study including 408 patients treated with interstitial brachytherapy, reporting five-year local control, penile preservation, and overall survival rates of 86%, 85%, and 82%, respectively. These results confirm the efficacy of brachytherapy as an organ-preserving strategy, reinforcing that conservative approaches can achieve outcomes comparable to surgery while maintaining quality of life [19].

HDR brachytherapy, in particular, has shown excellent functional and oncological outcomes. Marbán et al. (2019) reported that 82% of patients retained sexual function after HDR treatment, and most recurrences were successfully managed with penectomy [7]. Similarly, Rouscoff et al. (2014) reported five-year relapse-free, cause-specific, and overall survival rates of 83%, 100%, and 78%, respectively, with preservation of urinary and sexual function [8]. Pohanková et al. (2023) confirmed these results in a cohort of 31 patients, with five- and 10-year local control rates of 80.7% and 68.3%, penile preservation probabilities of 80.6% and 62.1%, and 100% cause-specific survival. Only one patient developed radionecrosis, highlighting the favorable safety profile of HDR brachytherapy [19].

The advantages over surgery are clear: brachytherapy allows organ preservation, maintains urinary and sexual function, and provides acceptable local control, with the possibility of surgical salvage in case of recurrence. While surgery may offer slightly higher local control, HDR brachytherapy represents an attractive option for patients refusing mutilating procedures, due to its capacity to preserve the organ and its function [20]. Similarly, Garaz et al. (2024) reviewed both brachytherapy and EBRT in primary penile cancer, showing that HDR brachytherapy provides superior five-year local control and penile preservation rates compared to EBRT. Late adverse events, such as soft tissue necrosis (0-31%) and meatal stenosis (0-43%), were lower with HDR, highlighting the importance of careful patient selection, multidisciplinary management, and dosimetric planning to optimize outcomes and minimize toxicity [6].

Limitations remain, both in the literature and in our study. Most studies are retrospective, with heterogeneous populations and variable techniques. Cohorts are generally small, and follow-up periods may not capture late recurrences. Our report describes a single patient with relatively short follow-up, limiting the ability to draw definitive conclusions regarding long-term oncologic control.

To contextualize our experience, we present the clinical outcomes of our patient alongside previously published series of penile brachytherapy (Table 2). Despite these limitations, our experience demonstrates that HDR brachytherapy can be successfully implemented in low-resource settings, expanding the feasibility of organ-preserving strategies beyond high-income centers. This approach provides a practical, effective alternative for patients who would otherwise face mutilating surgery, highlighting its potential relevance in broader clinical contexts.

**Table 2:** Comparative Analysis of Clinical Outcomes from Published Penile Brachytherapy Series. HDR: High-dose-rate brachytherapy; LDR: Low-dose-rate brachytherapy; PDR: Pulsed-dose-rate brachytherapy

Authors	n	MFU (months)	Type	Dose (Gy)	5y-LRFS (%)	5y-OS (%)	Necrosis (%)	Stenosis (%)	PP (%)
Kiltie et al., 2000 [21]	31	62	LDR	63.5	81	69	8	44	75
De Crevoisier et al., 2009 [22]	144	68	LDR	65	80	26	29	72	-
Crook et al., 2009 [23]	67	48	PDR/LDR	60	87	12	9	88	-
Escande et al., 2017 [24]	201	128	PDR/LDR	65	82	79	21.4	24.8	77.1
Peter et al., 2011 [25]	10	20	HDR	54	100	0	0	-	100
Rouscoff et al., 2014 [8]	12	27	HDR	36/39	83	78	9	9	92
Kellas-Sleczka et al., 2019 [26]	76	76	HDR	42.8/48	66	77	2.6	1.3	69.5
Pohankova et al., 2019 [27]	26	85	HDR	51	83	92	4	4	73
Marbán-Orejas et al., 2020 [7]	7	90	HDR	38.4/53	86	100	43	43	86
Ka et al., 2025 [28]	408	76	LDR/PDR/HDR	50-65	86	82	34.3 (acute ≥G2)	18.1 (late)	85
Martz et al., 2021 [2]	29	72	HDR	35/38	86	73	10	7	79
The present study	1	18	HDR	25 (first) / 20 (salvage)	-	-	0	0	100

In Morocco, penile cancer remains a socially sensitive and often stigmatized condition, which contributes to a high rate of patient refusal of mutilating surgical procedures. Outside of international publications, interstitial brachytherapy is not yet widely implemented, and most national reports focus on cervical and prostate cancers, reflecting limited expertise in this modality. The present case represents an initial implementation and a valuable learning curve for HDR brachytherapy in penile cancer, providing important practical insights for the management of future cases in a context where conservative, organ-preserving approaches may improve patient acceptance and outcomes.

## Conclusions

In conclusion, this case demonstrates the safety and feasibility of HDR brachytherapy as a promising organ-preserving treatment for glans squamous cell carcinoma, particularly in patients who refuse mutilating surgery in resource-limited settings. Although this represents the first reported experience in Africa, the results remain preliminary and warrant long-term follow-up to fully assess oncologic efficacy. Additional studies and further case documentation are needed to refine treatment protocols, better characterize functional and oncologic outcomes, and inform conservative management strategies in similar contexts.
